# First assessment of the comparative toxicity of ivermectin and moxidectin in adult dung beetles: Sub-lethal symptoms and pre-lethal consequences

**DOI:** 10.1038/s41598-018-33241-0

**Published:** 2018-10-05

**Authors:** José R. Verdú, Vieyle Cortez, Juan Martinez-Pinna, Antonio J. Ortiz, Jean-Pierre Lumaret, Jorge M. Lobo, Francisco Sánchez-Piñero, Catherine Numa

**Affiliations:** 1 I.U.I. CIBIO, Universidad de Alicante, Alicante, E-03080 Spain; 2 Departamento de Fisiología, Genética y Microbiología. Universidad de Alicante, Alicante, E-03080 Spain; 3 Departamento de Química Inorgánica y Química Orgánica, Universidad de Jaén, Campus Las Lagunillas, Jaén, E-23071 Spain; 4 Université Paul Valéry Montpellier 3, Univ. Montpellier, EPHE, CNRS, IRD, CEFE UMR 5175, F34000, Université Paul-Valéry Laboratoire Zoogéographie, route de Mende, 34199 Montpellier, cedex 5 France; 5 Museo Nacional de Ciencias Naturales-CSIC, Departamento de Biogeografía y Cambio Global, José Abascal 2, Madrid, E-28006 Spain; 6 Departamento de Zoología, Universidad de Granada, Granada, E-18071 Spain; 7 IUCN-Centre for Mediterranean Cooperation, Marie Curie 22, Campanillas, Málaga, E-29590 Spain

**Keywords:** Agroecology, Ecophysiology

## Abstract

Among macrocyclic lactones (ML), ivermectin (IVM) and moxidectin (MOX) potentially affect all Ecdysozoan species, with dung beetles being particularly sensitive. The comparative effects of IVM and MOX on adult dung beetles were assessed for the first time to determine both the physiological sub-lethal symptoms and pre-lethal consequences. Inhibition of antennal response and ataxia were tested as two intuitive and ecologically relevant parameters by obtaining the lowest observed effect concentration (LOEC) values and interpolating other relevant toxicity thresholds derived from concentration-response curves (IC_50_, as the concentration of each ML where the antennal response is inhibited by half; and pLC_50_, as the quantity of ingested ML where partial paralysis was observed by half of treated individuals) from concentration-response curves. Both sub-lethal and pre-lethal symptoms obtained in this study coincided in that IVM was six times more toxic than MOX for adult dung beetles. Values of LOEC, IC_50_ and pLC_50_ obtained for IVM and MOX evaluated in an environmental context indicate that MOX, despite needing more time for its elimination in the faeces, would be half as harmful to dung beetles as IVM. This approach will be valuable to clarify the real impact of MLs on dung beetle health and to avoid the subsequent environmental consequences.

## Introduction

**Figure 1:**
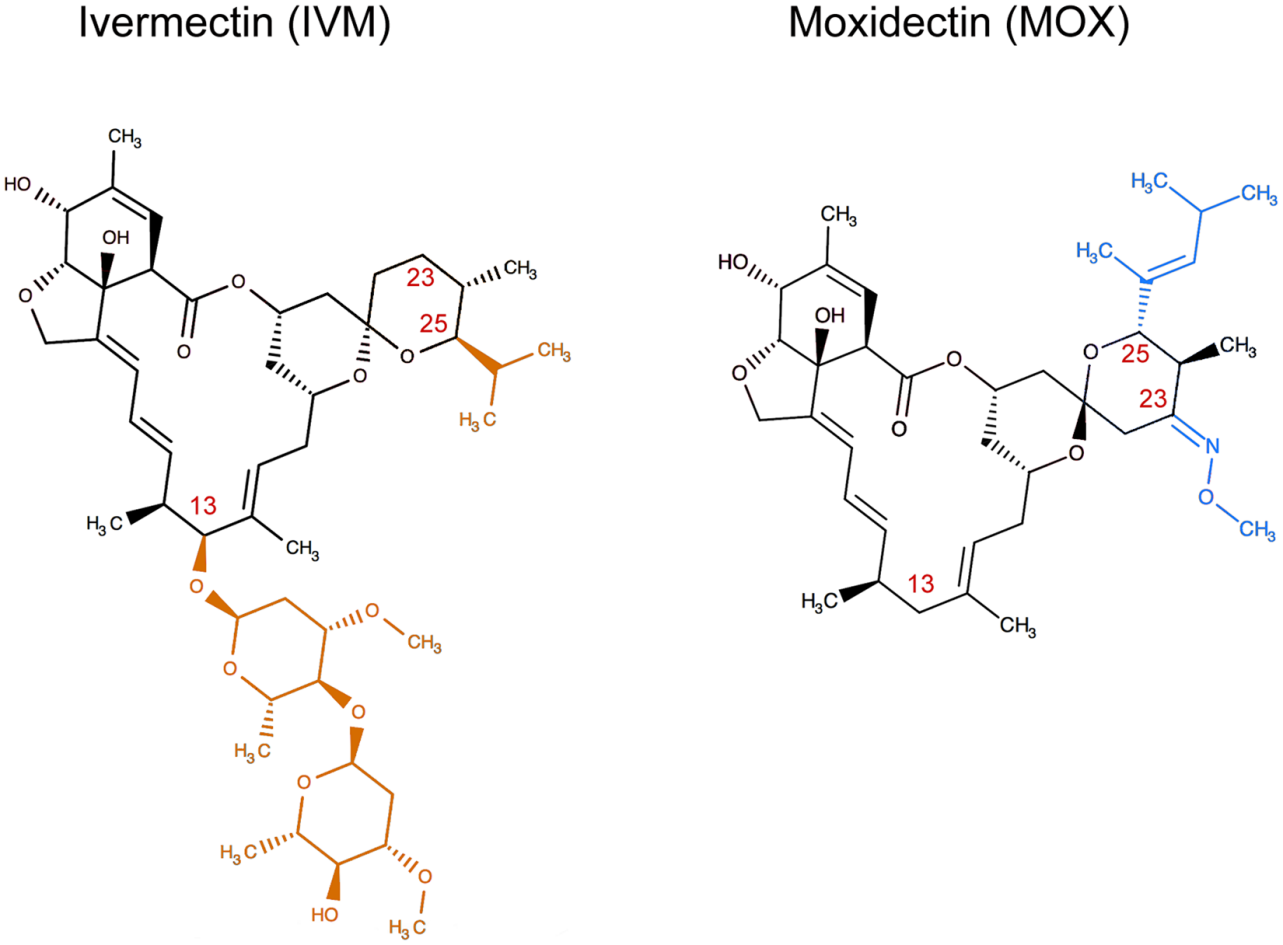
Comparison of the chemical structure of ivermectin (IVM) and moxidectin (MOX). The substituents that differ from IVM and MOX are highlighted in orange and blue, respectively. Red numbers indicate the C–positions.

Macrocyclic lactones (MLs) are a large family of broad-spectrum antiparasitic drugs derived from fermentation products of soil Actinomycetes: *Streptomyces avermitilis*, in the case of avermectins, and *S. cyanogriseus* for milbemycins. Ivermectin (IVM, an avermectin) and moxidectin (MOX, a milbemycin) are commonly used in veterinary medicine to treat livestock diseases caused by gastrointestinal worms, lung worms and ectoparasites, such as mites and blood-feeding insects. These two drugs differ in their chemical structure mainly in a disaccharide group, present in IVM and absent in MOX, and the presence of a 23-methoxyimino group in MOX and other specific substitutions^1^ (Fig. 1). As a consequence of these differences, IVM is a large, highly lipophilic molecule that is relatively insoluble in water, while MOX is considerably more lipophilic, which explains its longer mean residence time in the fat tissues of treated animals^2,3^. Despite these molecular differences, IVM and MOX’s modes of action are similar^1,4^, increasing plasma membrane permeability due to an agonistic action on chloride channels present in nerve and muscle cells. Both drugs act as positive allosteric regulators of several ligand-gated channels, including γ–aminobutyric acid (GABA)-gated chloride channels and glutamate-gated chloride channels (GluCl). GABA-gated chloride ion channels are present in neurons and abundant in local interneurons and antennal lobes of insects^5–8^ and are essential for olfactory processing. Furthermore, GABA_B_ receptors have been observed in olfactory sensory neurons in the male antenna of *Heliothis virescens* (F., 1777), initiating a GABA-mediated gain control mechanism that could play a pivotal role in processing pheromone signals^9^. Thus, toxic effects of IVM and MOX would manifest in insects as a reduction in sensorial response of antennae, paralysis and irreversible ataxia of somatic muscles, and death.

Due to their actions on both GluCl and GABA ion channels, IVM and MOX potentially affect all Ecdysozoan species as both target and non-target organisms^10^. Among the non-target organisms affected by these substances, dung beetles are particularly sensitive. Dung beetles are considered one of the most important groups within dung pat assemblages in terms of number of species, abundance, biomass and ecosystem services^11^. Given that the majority of the MLs administered as veterinary medical products (VMP) are excreted through cattle dung retaining their insecticidal activity^12^, a number of studies have been undertaken to assess the ecotoxicology of MLs on dung beetles. Although these studies are relatively numerous, there are limited comparative studies^13^ and/or studies using standardised toxicity thresholds^14^. Available evidence indicates that dung inhabiting organisms would have different sensitivity to these two VMP products, despite their similar modes of action^13,15^. For example, in dung flies (Muscidae, Scathophagidae and Sepsidae) mortality thresholds (LC_50_) were approximately ten times higher for MOX than for IVM; therefore, MOX is less toxic than IVM in absolute terms^14^. In the case of dung beetles, a comparative field study showed that the mortality of *Aphodius* spp. larvae was higher in dung collected from IVM-treated cattle for up to 7 days after treatment than in both MOX-treated and control dung^16^. Other studies reported that dung beetle species, such as *Digitonthophagus gazella* (F. 1787) and *Euoniticellus intermedius* (Reiche, 1849), showed reduced adult emergence when the dung originated from cattle treated with IVM, whereas the dung from cattle treated with MOX had no adverse effects on brood ball production and adult emergence^17,18^. In summary, dung beetles seem to be more sensitive to IVM than to MOX residues for both larval survival and brood ball production. However, it is necessary to corroborate the possible differential effect of these antiparasitic drugs on adults at both sub-lethal and pre-lethal levels.

In this study, the comparative effects of IVM and MOX on adult dung beetles were assessed for the first time examining both physiological sub-lethal symptoms and pre-lethal consequences after somatic paralysis. Following the same methodological guidelines proposed previously^19^, both the sensorial response of antennae (sub-lethal effect) and irreversible ataxia of somatic muscles (pre-lethal effect) were examined by exposing a model dung beetle species (*Scarabaeus cicatricosus* (Lucas, 1846); Coleoptera, Scarabaeidae) to both IVM and MOX under different dose concentrations.

## Results

**Figure 2:**
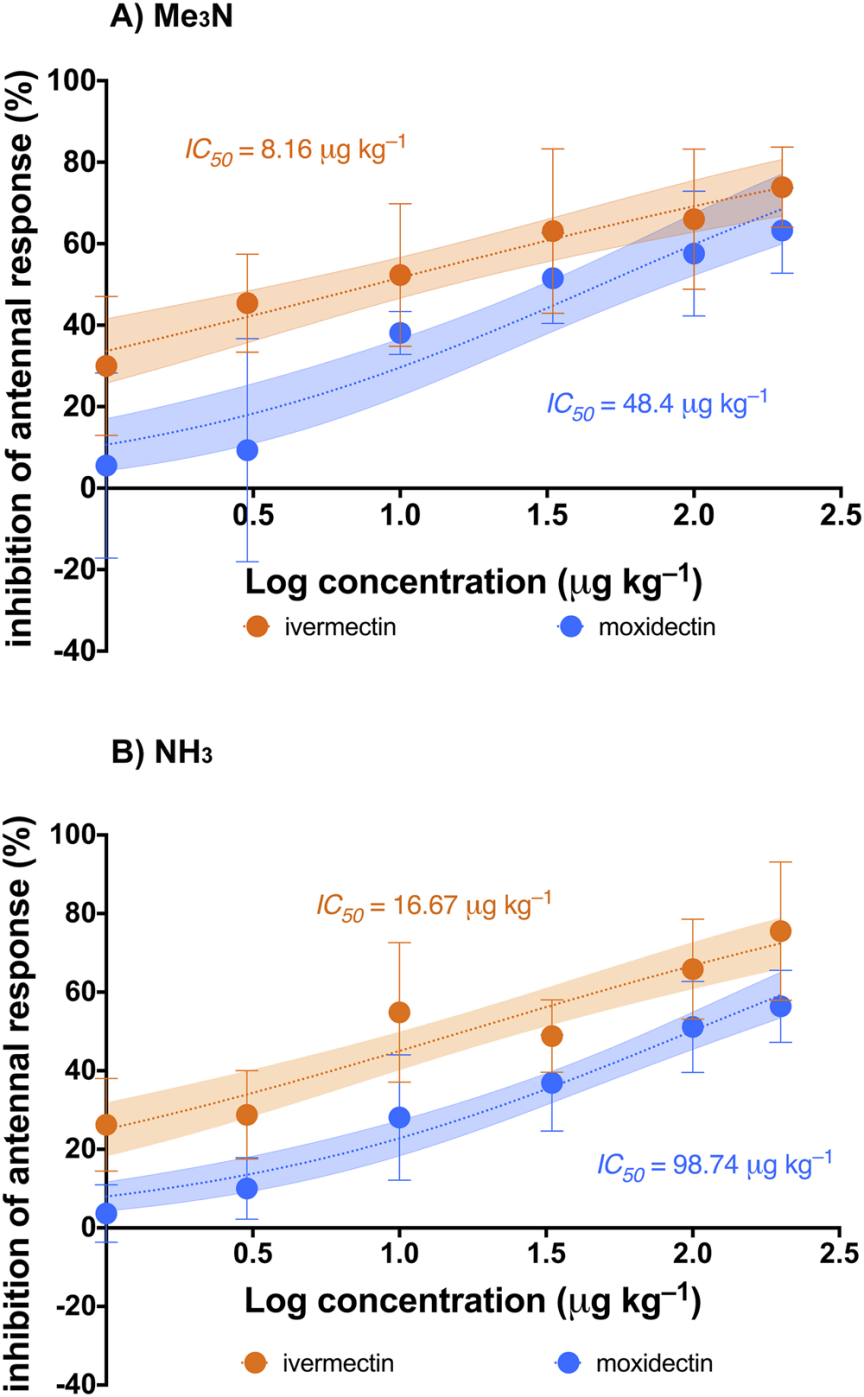
Concentration response curves for inhibition of antennal response by ivermectin (IVM) and moxidectin (MOX), using trimethylamine (**A**) and ammonia (**B**) as test odorants. Bars represent ± SD (n = 8). Shaded areas represent the 95% confidence intervals of each model. IC_50_ corresponds to the concentration of each ML that inhibited 50% of antennal response. Statistical results of the effect concentrations (IC_50_) by MLs for each odorant are provided in Table 1.

Electroantennography recordings showed that both IVM and MOX ingestion negatively but differently affected the antennal olfactory apparatus of *S. cicatricosus*. For both tested odorants, LOECs obtained were 1.0 µg kg^−1^ (fw) and 10.0 µg kg^−1^ (fw) for both IVM and MOX, respectively (Table 1). Fitting the % inhibition of antennal response data to log (inhibitor) vs. normalised response models allowed for the interpolation of concentrations of both VMPs that inhibited the antennal response in *S. cicatricosus* adults by 50% (IC_50_) (*P* < 0.05; Table 1; Fig. 2). Using the Me_3_N odorant, IC_50_ was 8.16 µg kg^−1^ (fw) and 48.40 µg kg^−1^ (fw) for IVM and MOX, respectively (*P* < 0.05; Fig. 2A). The EAG test performed with NH_3_ yielded an IC_50_ of 16.67 µg kg^−1^ (fw) and 98.74 µg kg^−1^ (fw) for IVM and MOX, respectively (*P* < 0.05; Fig. 2B). Thus, for both odorants, IVM was six times more toxic than MOX for adult *S. cicatricosus*.

**Table 1:** Concentrations of IVM and MOX where the antennal response of adults of *Scarabaeus cicatricosus* is inhibited by half (IC_50_) (95% CV intervals), calculated from dose-response curves presented in Fig. 2, and lowest significant observed effect concentration (LOEC) reported from Dunnet’s tests performed in the ANOVA-GLM analyses.

Test	Ivermectin	Moxidectin
**EAG inhibition (Me**_**3**_ **N)**		
IC_50_ (CI 95%) (µg kg^−1^)	8.16 (*3.94–15.03*)	48.40 (*30.24–83.17*)
ANOVA *F* (DFn, DFd)	16.60 (1, 46)	95.87 (1, 46)
*P*	<0.001	<0.0001
LOEC (µg kg^−1^)	1.00	10.00
ANOVA *F* (DFn, DFd)	14.37 (6, 49)	16.31 (6, 49)
*P*	<0.0001	<0.0001
**EAG inhibition (NH**_**3**_ **)**		
IC_50_ (CI 95%) (µg kg^−1^)	16.67 (*10.14–27.26*)	98.74 (*70.82–147.80*)
ANOVA *F* (DFn, DFd)	46.81 (1, 46)	270.50 (1, 46)
*P*	<0.0001	<0.0001
LOEC (µg kg^−1^)	1.00	10.00
ANOVA *F* (DFn, DFd)	30.69 (6, 49)	34.45 (6, 49)
*P*	<0.0001	<0.0001

**Figure 3:**
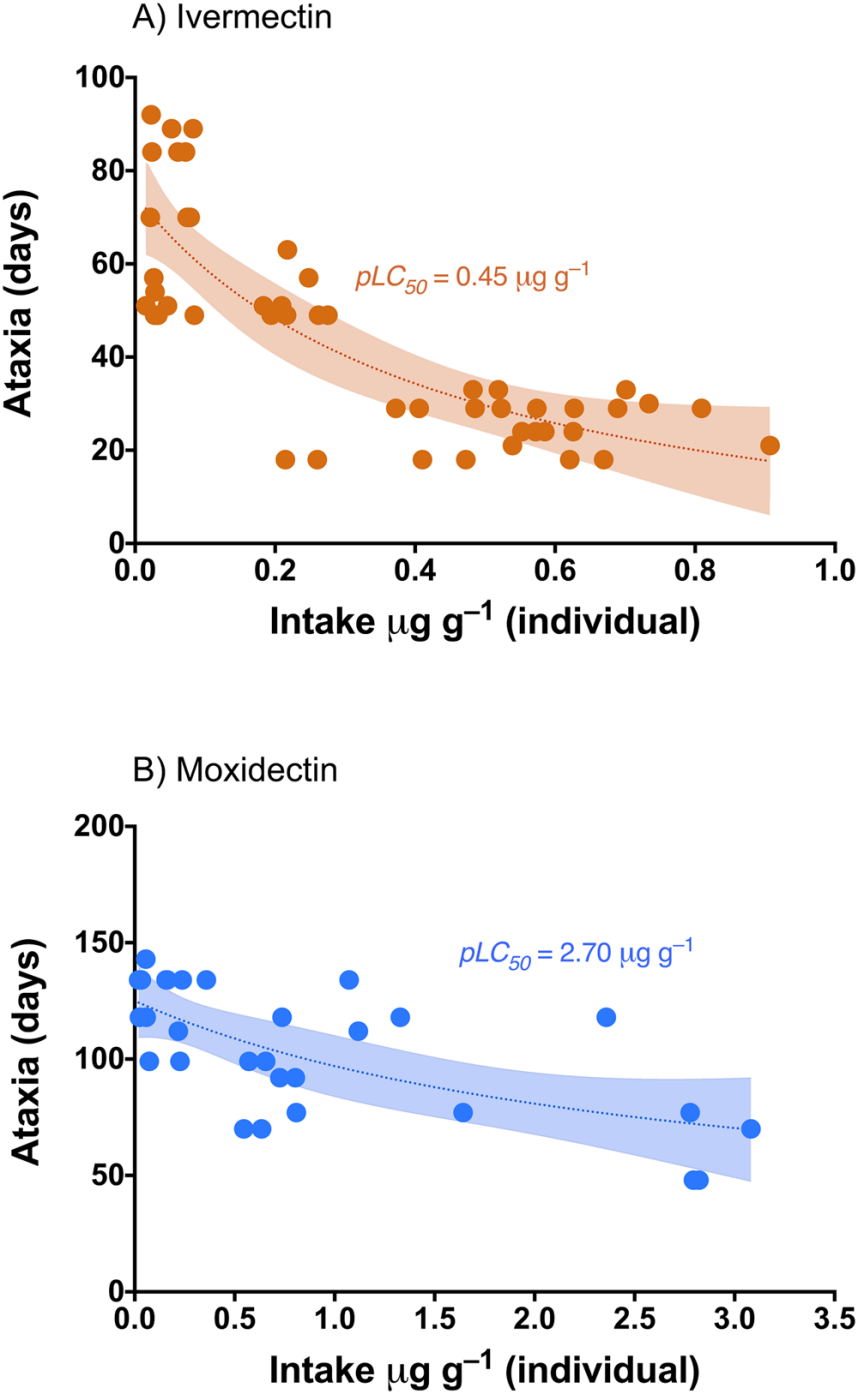
Total ingestion response curves for ataxia symptoms by ivermectin (IVM) (**A**) and moxidectin (MOX) (**B**). Shaded areas represent the 95% confidence intervals of each model. pLC_50_ corresponds to the quantity of each ML ingested by beetles (µg of each ML/g of individual beetles) that provoked a response halfway between the minimum number of days and the maximum number of days that produced pre-lethal paralysis.

In the pre-lethal test based on ataxia symptoms, we also observed notable differences between the two studied MLs. First, laboratory observations showed that all beetles treated with IVM reached paralysis in relation to the quantity of drug ingested (dose), while some of the beetles that were treated with MOX did not suffer ataxia (37.5% of the total). Fitting the data for time duration until ataxia to three parameter inhibitor vs. response models allowed for the interpolation of the dose of both MLs that caused paralysis and subsequent death in *S. cicatricosus* adults by 50% (pLC_50_). In both cases, the Runs test showed that data were significantly adjusted to the model (IVM: *P* = 0.10; MOX: *P* = 0.43). For IVM, pLC_50_ was 0.45 µg g^−1^, while for MOX, pLC_50_ was 2.70 µg g^−1^, demonstrating that IVM was six times more toxic than MOX (Table 1; Fig. 3).

## Discussion

Both sub-lethal and pre-lethal symptoms obtained in this study coincide in that IVM is six times more toxic than MOX for adults of *S. cicatricosus*. This is the first study to examine the comparative effects of IVM and MOX on the physiology of adult dung beetles using electroantennography procedures. Sub-lethal effects, such as those measured in this study, could imply that mature beetles feeding on dung, even at low concentrations of IVM and MOX, may experience an acute toxicity that would prevent the performance of normal biological activities, such as food detection, intraspecific communication, locomotion and interaction with the environment^19^. This research also represents one of notably few studies on ML ecotoxicology that incorporates significant toxicological values such as the generation of dose-response curves at different concentrations (e.g., to calculate LOEC and IC_50_ values), which will allow for the comparison of ecotoxicity between different molecules in future studies aiming to establish risk assessments of veterinary medicines. Even though the effects of individual MLs on dung beetle species have been previously studied^13^, their results are difficult to compare because of the lack of a standardised and common methodological procedure. Literature comparing IVM and MOX toxicological effects shows that test results reported in this study are approximately on the same order of magnitude as those determined by other studies. For example, in a test on larvae of the dung beetle *Agrilinus constans* (Duftschmid, 1805) (reported in older literature as *Aphodius constans* Duft.) using mortality thresholds (LC_50_), IVM was five times more toxic than MOX^20^. In a related study that tested the LC_50_ of the larval stages for 11 dung fly species (Diptera), IVM was approximately ten times more toxic than MOX^14^.

Although the differences between the toxicity of the two MLs were significant for all studied variables, values of LOEC, IC_50_ and pLC_50_ obtained for IVM and MOX should be evaluated in an environmental context to discern whether dose thresholds are appreciably lower than those usually detected in the dung of treated livestock. Considering the different pharmacokinetics and metabolic behaviour of the two MLs, two days after cattle treatment (coinciding with the most frequent peak level of residue excretion), 100.8 μg kg^−1^ (fw) of IVM and 43.6 μg kg^−1^ of MOX (fw) were measured in the field (subcutaneous injection: 300 μg kg^−1^ bw and 200 μg kg^−1^ bw, for IVM and MOX, respectively)^21^. The faecal excretion profiles of IVM and MOX vary according to differences in the pharmacokinetic, metabolic behaviour of livestock, the supply method, dosage and diet^22^. However, in general terms, the IVM concentration is still relatively high after two weeks (~86.5 μg kg^−1^ fw); even after 28 days, IVM was still detectable at ~6.1 μg kg^−1^ (fw)^23^. In the case of MOX, excretion of residues in faeces continues for more than 28 days, at which time concentrations are relatively low at ~5.0 μg kg^−1^ (fw)^21^. According to our results, MOX residues observed in the field are below the LOEC value obtained (LOEC = 10.0 μg kg^−1^) after the second week of treatment, while IVM concentrations close to the LOEC level (LOEC = 1.0 μg kg^−1^) are not observed until after at least 28 days^24^. Thus, from an environmental point of view, obtained LOEC values indicate that MOX, despite needing more time for its elimination in the faeces could be half as harmful to dung beetles as IVM. Furthermore, a comparison of the toxic thresholds (IC_50_) derived from our laboratory study (see Table 1) against concentrations (peak of residue excretion) of MLs measured in the field^21^, shows that the IC_50_ for MOX is greater than that obtained in the field, indicating that mature *S. cicatricosus* could be at an even lower risk. Conversely, in the case of IVM, obtained IC_50_ values are lower than those corresponding to the peaks of excretion obtained in some field studies^25,26^, thus suggesting an increased environmental risk for dung beetles.

Also of considerable interest was our finding that in a number of cases, dung beetles have no symptoms of apparent muscular paralysis (37.5% of the total treated), despite being fed dung treated with MOX. This result concurs with the results of the relationships between the time required to produce ataxia and the intake doses of MOX and IVM (Fig. 3). In the case of MOX, the lower toxicity observed in the antennal response test implies that the quantity of drug ingested by a mature beetle necessary for the attainment of ataxia symptoms (see pLC_50_ values in Table 1) should be significantly higher than for individuals feeding on dung containing IVM. These results are also in agreement with those obtained with *A. constans*, whose females do not stop laying eggs even in the presence of high concentrations of MOX that are likely to kill their offspring^27^. Furthermore, Doherty *et al*.^28^ estimated that in cattle dung, MOX was approximately 64 times less toxic to fly and beetle larvae than abamectin and concluded that MOX was unlikely to have deleterious effects on dung fauna when used in accordance with the manufacturer’s instructions.

The differences observed in sub-lethal and pre-lethal thresholds between IVM and MOX treatments could be related to differences in the molecular structure of both MLs (see Fig. 1). These differences, such as the absence of the bisoleandrosyl moiety in the C-13 position of the macrocycle and the existence of a methoxime moiety at C-23 and an olefinic side chain at C-25 in MOX (Fig. 1), could be postulated as responsible for the different interactions of both MLs with glutamate-gated and GABA-gated chloride channels^1^. Although the mechanisms for such differences remain unknown, neurotoxicity signs observed in mice showed that IVM is 5 times more toxic, causing an almost 2-fold maximum potentiation of the GABA_A_ receptor and potentiating the effects of GABA binding and opening of channels compared with MOX at similar concentrations^29^. Moreover, based on a model for the IVM binding site and atomic interactions with amino acids observed in *Caenorhabditis elegans* (Maupas)^30^ and considering the structural differences described above, studies have postulated that the interaction of MOX with both glutamate-gated chloride channels and GABA-gated chloride channels will be different from that of IVM^1^. When we analysed the effects of IVM and MOX on the EAG signals, we observed that both the peak amplitude and the area under the curve of the EAG signal were affected similarly by both MLs. However, the latency to the peak of the EAG was unaffected by both treatments, suggesting that all types of nerve fibres (fast and slow) were equally affected by both MLs (unpublished observations).

In conclusion, the results obtained in this study demonstrate that adult dung beetles are significantly more susceptible to IVM than to MOX ingestion. Detectable sub-lethal effects using physiological and behavioural tests performed with dose-response curves are crucial to accurately compare ecotoxicological effects of different MLs. Definitive tests performed here with appropriate statistical analysis to determine point estimates, such as LOEC, IC_50_ and pLC_50_, are recommended to evaluate chronic toxicity in an environmental context as a useful tool for the risk assessment of veterinary medicines. Given that MLs are currently used worldwide with registrations in over 60 countries^13^, comparative and standardised tests regarding acute and chronic ecotoxic properties of MLs are necessary to update the registration dossiers of companies and regulate their use in order to minimise the negative effects on non-target organisms. In the European Union, the international and European guidelines^31–33^ and previous Environmental Risk Assessments (ERA) conducted on IVM revealed no concern for the aquatic compartment of ecosystems, and transient effects on the dung-insect community were not considered relevant. However, a more recent ERA^34^ demonstrated serious risks for all analysed environmental compartments, suggesting the necessity of reassessing IVM-containing products by medicine agencies, such as the European Medicines Agency (EMEA). In the case of MOX, the EMEA’s Committee for Medicinal Products for Veterinary Use (CVMP) determined that MOX might have a long-term impact on the environment due to its persistent, bioaccumulative and toxic (PBT) properties^35,36^. Based on these gaps in knowledge and possible incongruences, EMEA’s field of activity clearly requires advanced technical and scientific knowledge. Thus, we suggest the implementation of comparative physiological and behavioural testing in further ecotoxicological laboratory standardised tests required by the International Cooperation on Harmonisation of Technical Requirements for Registration of Veterinary Medicinal Products (VICH). This approach will be valuable to clarify the real impacts of MLs on dung beetle health and to avoid the subsequent environmental consequences, considering the crucial role of this group of insects in the complex process of faecal degradation in grazed ecosystems.

## Methods

### Collection, selection and preparation of beetles

The target species, *Scarabaeus cicatricosus*, was selected because it has well established populations in the site of study (Doñana Biological Reserve, DBR-ICTS), an ivermectin-free site within the Doñana National Park (Huelva) in southern Spain. *S. cicatricosus* is a dung beetle with a large body size (~25 mm) distributed in the southern Atlantic areas of the Iberian Peninsula and Morocco in sandy coastal soils. The high abundance (it is possible to collect more than 30 individuals per dung pat) and biomass of this beetle species (approximately 1.5 g of fresh weight) make it a keystone species from the functional point of view. For these reasons, we decided to use ‘healthy’ specimens of this species collected under uncontaminated localities to examine the comparative effects of ingesting ivermectin and moxidectin.

Individuals of *S. cicatricosus* were collected from the DBR-ICTS during the summer (July 2016). All individuals were maintained in plastic containers (60 × 40 × 40 cm) at 20 °C prior to arrival at the laboratory, where they were sustained in a climate chamber at 29** ± **1: 21** ± **1 °C (L: D), 80 ± 5 RH with a photoperiod of 14:10 (L: D). These conditions were similar to the optimal conditions experienced in the field^37^.

To maximise a common physiological state for all the individuals, only mature specimens were selected according to external age-grading methods (e.g., tibial and clypeal wear) that allow for the identification of individuals of approximately the same age^38^. In addition, we used a 1:1 sex ratio in each experiment. This work conforms to the Spanish legal requirements, including those relating to conservation and welfare. Additionally, beetle collection was conducted with relevant permissions related to collection and field study within Doñana National Park.

### Preparation of dung treatments

Non-contaminated bovine dung was obtained from VMP-free cattle in the Doñana Biological Reserve. Fresh dung was collected from 06:00 to 08:00 AM to avoid dung colonisation by insects as well as to minimise physical-chemical changes in the dung. If not used immediately, dung was cooled (3 °C) until its usage.

Dung (~20 kg) was homogenised using an electric paint mixer. Subsequently, an untreated control and six IVM and MOX concentrations were selected according to literature^13,14^ and a previous study with the same species^19^: 1.0, 3.3, 10.0, 33.3, 100.0, and 200.0 μg kg^−1^ (fresh weight) allowing us to compare a wide range of doses on a single analysis. Solutions were made by dissolving IVM (Merck KGaA, Darmstadt, Germany) and MOX (Merck KGaA, Darmstadt, Germany) in absolute ethanol (Merck KGaA, Darmstadt, Germany), and 2 ml aliquots of each drug in the six selected concentrations were added to 2 kg portions of fresh dung, mixing all for 2 h by means of a kitchen machine mixer. For the untreated control, absolute ethanol (2 ml) was applied to the same quantity of dung. Residual ethanol was removed during evaporation for 6 h before transferring the dung treatments to individual experimental units.

### Bioassay design

Each individual experimental unit consisted of a 15 × 10 × 7 cm plastic container using moist vermiculite as substrate. Dung treatments were supplied in 4 ml portions on a 6 cm petri dish avoiding contact with the substrate to better quantify the amount of IVM and MOX ingested per individual. Every three days, the unconsumed dung was removed and measured (in ml), adding a new portion of the corresponding dung treatment. In the case of the electroantennogram (EAG) tests, beetles in each treatment were fed with treated dung for an average of 12 days before conducting the bioassays. For EAG and ataxia tests, each treatment was replicated eight times. Beetles were sexed, numbered and weighed (fresh body mass) prior to assignment to each treatment.

### Olfactory extracellular recordings

Electroantennogram bioassays were performed using an EAG system (Syntech, Kirchzarten, Germany) consisting of an EAG probe containing pre-amplifier (Type PRG-2), a data acquisition interface board (Type IDAC-2), and a stimulus air controller (Type CS-55). Based on a previous protocol^19^, the antennae of each individual *S. cicatricosus* were excised and mounted between two metal electrodes in an antenna holder using small droplets of conductive gel (Spectra 360, Parker Laboratories, Fairfield, NJ, USA) and placed under a constant stream of humidified air (flow of 500 ml min^−1^). A Syntech PC-based signal processing system was used to amplify and process the EAG signals. The signals were further analysed using EAG 2000 software (Syntech, Kirchzarten, Germany). An EAG signal is the algebraic sum of all individual odorant receptor action potentials of the antennae.

Stimulation tests were conducted by applying puffs of humidified air (200 ml/min) flowing for 2 s using the CS-55 stimulus controller through a Pasteur pipette containing a small strip (1 cm^2^) of filter paper (Whatman no. 1) with 1 μl of one of the test compounds flowing through a stainless steel delivery tube (1 cm diameter) with the outlet positioned approximately 1 cm from the antenna. In each experiment, the antenna was first presented with an injection of the standard reference compound, hexane (HPLC grade, Sigma-Aldrich Co.), and then with injections of test odorants. Based on a previous study^19^, standards of ammonia aqueous (25% NH_3_ in H_2_O, CAS Number 1336-21-6, Sigma-Aldrich Co.) and trimethylamine (Me_3_N, 25 wt. % in H_2_O, CAS Number 75-50-3, Sigma-Aldrich Co.) were selected as test odorants. Puffs of the tested compounds were applied at 1 min intervals at least 10 times on each antenna. Replicates were performed with different individuals (*n* = 8, for each treatment). There was no reduction in the response of the reference stimulus throughout the tests in any of the replicates.

### Ataxia test

For each individual, we supplied 4 ml of dung (either control or treated dung). Every three days, possible negative symptoms related to each VMP ingestion were assessed by performing two observations: (a) coordinated walking and (b) reflex avoidance movements of the scape-pedicel joint of the antenna. When a normal behaviour was observed, we concluded that the beetle was healthy. However, if partial paralysis was observed in the legs and/or antennae, we recorded the date of observation and described the symptoms. This assay was recorded each three days until no symptoms were observed in the surviving individuals (final time = 165 days). Each treatment was replicated eight times (*n* = 8 individuals/treatment).

### Data analyses

The inhibition of antennal response was selected as a tested sensitive and ecologically relevant parameter, interpolating the toxicity threshold (IC_50_, as the concentration of each ML where the antennal response is inhibited by half) from concentration-response curves. Inhibition of antennal response (% inhibition relative to EAG_control_) was calculated from EAG peak amplitude data as Inhibition (%) = 100 × ((EAG_control_ − EAG_treatment_)/EAG_control_). The drug concentration that inhibited 50% of the antennal response (IC_50_) was calculated from concentration-response curves (log (inhibitor) vs. normalised response models) fitted to the percentage of inhibition of each treatment using the programme GraphPad Prism (v7, San Diego, USA). Applying the *F* test in GraphPad Prism v7 tested the probability that IC_50_ values generated by the logistic curves were significantly different between both drugs (*P* < 0.05). Furthermore, the lowest observed effect concentration (LOEC) was determined by an ANOVA design using General Linear Models (GLM). Normality was examined using a Kolmogorov-Smirnov test (*P* > 0.05 in all cases). Multiple post hoc comparisons between treatment groups against the control group were made using Dunnet’s tests (EAG test: mean of treatment < mean of control). The software STATISTICA v8.0 (StatSoft Inc, Tulsa, Oklahoma, USA) was used for these statistical analyses.

For the pre-lethal test (ataxia), we calculated the total ingestion of each ML until irreversible paralysis of each individual, normalising it by fresh body weight (µg ML g^−1^ individual). In these analyses, we used individual data (not replicated data). Data sets for each ML were fitted to a three parameter inhibitor vs. response model. The ‘Runs test’ was performed to test whether the curve deviated systematically from our data. We calculated the quantity (µg g^−1^) of each ingested ML that induced a response of halfway between the minimum and maximum number of days that produced pre-lethal paralysis (pLC_50_) using the software GraphPad Prism v7.

## Data Availability

The datasets analysed during the current study are available from the corresponding author on reasonable request.
