# Author Correction: First assessment of the comparative toxicity of ivermectin and moxidectin in adult dung beetles: Sub-lethal symptoms and pre-lethal consequences

**DOI:** 10.1038/s41598-019-43806-2

**Published:** 2019-05-21

**Authors:** José R. Verdú, Vieyle Cortez, Juan Martinez-Pinna, Antonio J. Ortiz, Jean-Pierre Lumaret, Jorge M. Lobo, Francisco Sánchez-Piñero, Catherine Numa

**Affiliations:** 1 I.U.I. CIBIO, Universidad de Alicante, Alicante, E-03080 Spain; 2 Departamento de Fisiología, Genética y Microbiología. Universidad de Alicante, Alicante, E-03080 Spain; 3 Departamento de Química Inorgánica y Química Orgánica, Universidad de Jaén, Campus Las Lagunillas, Jaén, E-23071 Spain; 4 Université Paul Valéry Montpellier 3, Univ. Montpellier, EPHE, CNRS, IRD, CEFE UMR 5175, F34000, Université Paul-Valéry Laboratoire Zoogéographie, route de Mende, 34199 Montpellier, cedex 5 France; 5 Museo Nacional de Ciencias Naturales-CSIC, Departamento de Biogeografía y Cambio Global, José Abascal 2, Madrid, E-28006 Spain; 6 Departamento de Zoología, Universidad de Granada, Granada, E-18071 Spain; 7 IUCN-Centre for Mediterranean Cooperation, Marie Curie 22, Campanillas, Málaga, E-29590 Spain

Correction to: *Scientific Reports* 10.1038/s41598-018-33241-0, published online 05 October 2018

The original version of this Article contained typographical errors in the Abstract.

“Values of LOEC, IC_50_ and pLC_50_ obtained for IVM and MOX evaluated in an environmental context indicate that MOX, despite needing more time for tis elimination in the faeces, would be twice as harmful to dung beetles as IVM.”

now reads:

“Values of LOEC, IC_50_ and pLC_50_ obtained for IVM and MOX evaluated in an environmental context indicate that MOX, despite needing more time for its elimination in the faeces, would be half as harmful to dung beetles as IVM.”

Furthermore, in the second paragraph of the Discussion section:

“Thus, from an environmental point of view, obtained LOEC values indicate that MOX, despite needing more time for its elimination in the faeces could be twice as harmful to dung beetles as IVM.”

now reads:

“Thus, from an environmental point of view, obtained LOEC values indicate that MOX, despite needing more time for its elimination in the faeces could be half as harmful to dung beetles as IVM.”

These errors have now been corrected in the PDF and HTML versions of the Article.

